# The Aquatic Symbiosis Genomics Project: probing the evolution of symbiosis across the Tree of Life

**DOI:** 10.12688/wellcomeopenres.17222.2

**Published:** 2024-06-10

**Authors:** Victoria McKenna, John M. Archibald, Roxanne Beinart, Michael N. Dawson, Ute Hentschel, Patrick J. Keeling, Jose V. Lopez, José M. Martín-Durán, Jillian M. Petersen, Julia D. Sigwart, Oleg Simakov, Kelly R. Sutherland, Michael Sweet, Nicholas J. Talbot, Anne W. Thompson, Sara Bender, Peter W. Harrison, Jeena Rajan, Guy Cochrane, Matthew Berriman, Mara K.N. Lawniczak, Mark Blaxter

**Affiliations:** 1 Tree of Life, Wellcome Sanger Institute, Cambridge, CB10 1SA, UK; 2 Department of Biochemistry and Molecular Biology, Dalhousie University, Halifax, Nova Scotia, B3H 4R2, Canada; 3 Graduate School of Oceanography, University of Rhode Island, Narragansett, Rhode Island, 02882, USA; 4 Life & Environmental Sciences, University of California, Merced, Merced, California, 95343, USA; 5 Marine Symbioses Research Unit, GEOMAR Helmholtz Centre for Ocean Research, Kiel, Germany; 6 Department of Botany, University of British Columbia, Vancouver, British Columbia, V6T 1Z4, Canada; 7 Halmos College of Arts and Sciences, Nova Southeastern University, Dania Beach, Florida, 33004, USA; 8 School of Biological and Chemical Sciences, Queen Mary University of London, London, E1 4NA, UK; 9 Centre for Microbiology and Environmental Systems Science, University of Vienna, Vienna, A-1090, Austria; 10 Marine Zoology Department, Senckenberg Research Institute, Frankffurt, 60325, Germany; 11 Department for Neurosciences and Developmental Biology, University of Vienna, Vienna, 1010, Austria; 12 Department of Biology, University of Oregon, Eugene, Oregon, 97403-1210, USA; 13 Aquatic Research Facility, Environmental Sustainability Research Centre, University of Derby, Derby, DE22 1GB, UK; 14 The Sainsbury Laboratory, Norwich, NR4 7UH, UK; 15 Department of Biology, Portland State University, Portland, Oregon, 97201, USA; 16 Gordon and Betty Moore Foundation, Palo Alto, California, 94304, USA; 17 European Molecular Biology Laboratory, European Bioinformatics Institute, Cambridge, CB10 1SD, UK

**Keywords:** Symbiosis, Marine, Freshwater, Genome Sequencing, Collaboration, Open Science

## Abstract

We present the Aquatic Symbiosis Genomics Project, a global collaboration to generate high quality genome sequences for a wide range of eukaryotes and their microbial symbionts. Launched under the Symbiosis in Aquatic Systems Initiative of the Gordon and Betty Moore Foundation, the ASG Project brings together researchers from across the globe who hope to use these reference genomes to augment and extend their analyses of the dynamics, mechanisms and environmental importance of symbioses. Applying large-scale, high-throughput sequencing and assembly technologies, the ASG collaboration will assemble and annotate the genomes of 500 symbiotic organisms – both the “hosts” and the microbial symbionts with which they associate. These data will be released openly to benefit all who work on symbioses, from conservation geneticists to those interested in the origin of the eukaryotic cell.

## Disclaimer

The views expressed in this article are those of the author(s). Publication in Wellcome Open Research does not imply endorsement by Wellcome.

## The genomics of symbiosis

**Figure 1.**
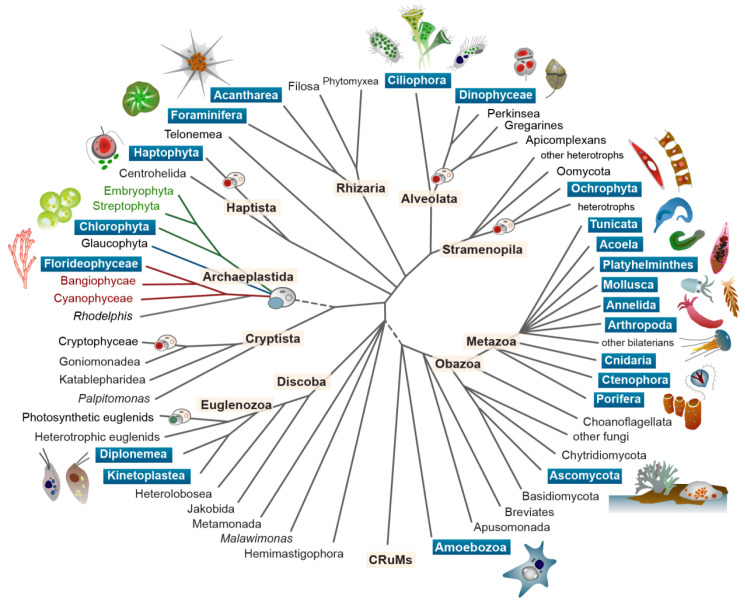
The phylogenetic diversity of eukaryotic symbioses. Symbiotic taxa, and Aquatic Symbiosis Genomics target species, are found across the diversity of the eukaryotic Tree of Life. Taxa highlighted with blue boxes include ASG targets. Within the tree, the small cartoons indicate the major event of plastid acquisition through symbiosis with a cyanobacterium (in the Archaeplastida; blue cell engulfed) and the several events of secondary and tertiary plastid acquisition in other lineages. The taxa containing primary plastids are shown in green and red. Illustration by John Archibald and Mark Blaxter.

Symbiosis, the living together of distinct organisms ( Archibald, 2014; Oulhen *et al*., 2016), describes a spectrum of relationships from mutualistic to parasitic, and from obligate to temporary. Symbiosis has been and is fundamental to the evolution of life on Earth, from the deep origins of the eukaryotic cell and photosynthetic eukaryotes, through to the recent emergence of new partnerships. The power of symbiosis arises from the ability of the joint organism to draw from the independent, billion-year evolutionary histories of both partners. Symbiosis is a fact of life – it has arisen many, many times and new symbioses are constantly evolving ( Figure 1). In this era of rapid climate change and biodiversity loss, many keystone symbiotic systems are threatened, and their loss imperils the ecosystems they support.

Well-known mutualist symbioses permit colonisation of otherwise inaccessible habitats, are critical to ecosystem functioning, and support marine and freshwater diversity. For example, coral reefs, built through a photosymbiotic association between cnidarians and dinoflagellate algae ( LaJeunesse *et al*., 2018; Weis, 2019), create biodiversity hotspots which house upwards of 25% of all described ocean species ( Fisher *et al*., 2015). The dominant animals colonising deep-sea hydrothermal vents are nutritionally dependent on chemosymbiotic associations with bacteria ( Roeselers & Newton, 2012), allowing them to thrive in the food-limited dark ocean. For these symbioses, the biological fitness consequences are largely understood, but in many less well-known symbioses, such as those between sponges and their bacterial collaborators, or partnerships in the diverse world of single celled eukaryotes, the basis of the relationships are not known in any detail.

## The aquatic symbiosis genomics project will transform symbiosis research

The Gordon and Betty Moore Foundation has created a major funding initiative focused on investigating the biology of symbiosis in marine and freshwater ecosystems (see Symbiosis in Aquatic Systems Initiative). To support this global initiative, the Aquatic Symbiosis Genomics project (ASG; see Aquatic Symbiosis Genomics Project – Wellcome Sanger Institute) plans to generate high-quality genome sequences from a wide range of symbiotic systems. Our focus is on symbioses involving at least one eukaryotic partner, and where there is likely to be co-evolving interplay between the species involved.

Like a symbiotic organism, the ASG project is more than the simple sum of its parts. ASG will merge the decades of ecological, evolutionary, taxonomic, and experimental expertise of researchers from diverse backgrounds with the decades of genomics experience of the Wellcome Sanger Institute. ASG works on a hub and spokes model, where communities of researchers nucleated on specific questions and/or species systems have come together as hubs to propose sets of taxa for sequencing ( Table 1). These (currently) total ~450 distinct symbiotic organisms from the open ocean, the deep sea, coastal, littoral, and freshwater ecosystems, which are expected to include over 1000 nominal species of hosts and symbionts. The ASG target list includes species representing many phyla of animals, protists, algae and fungi, and encompasses ancient and recently-evolved partnerships.

**Table 1.** Aquatic symbiosis genomics project hubs.

Lead researcher *	*Project Title (short)*	*Major taxa represented*	
		*Hosts*	*Symbionts*
**Archibald**	New symbioses in single-celled eukaryotes	Amoebozoa, Dinophyceae, Diplonemea (Euglenozoa), Haptophyta, Ochrophyta	Bacteria, Kinetoplastea, Ochrophyta
**Beinart, Petersen,** ** Sigwart**	Molluscan symbioses	Mollusca	Arthropoda, Bacteria, Chlorophyta, Cnidaria, Dinophyceae, Platyhelminthes, Florideophyceae
**Dawson, Sutherland,** ** Thompson**	Pelagic symbioses	Acoela, Ctenophora, Cnidaria, Tunicata	Bacteria, Chlorophyta, Dinophyceae, other Alveolata
**Hentschel**	Sponge symbioses	Porifera	Bacteria, Archaea, Viruses, Symbiodiniaceae (Dinophyceae) and others
**Keeling**	Symbiosis in ciliates	Ciliophora	Archaea, Bacteria, Chlorophyta, Ciliophora, Dinophyceae
**Lopez**	Metazoan photosymbioses	Acoela, Cnidaria, Mollusca, Porifera, Tunicata	Bacteria, Chlorophyta, Cnidaria, Dinophyceae, Haptophyta, Myzozoa
**Martín-Durán**	Annelid chemosymbioses	Annelida	Bacteria, Archaea
**Simakov**	Cephalopod symbioses	Mollusca	Bacteria, Archaea
**Sweet**	Coral symbioses	Cnidaria	Symbiodiniaceae (Dinophyceae)
**Talbot**	Marine lichens	Fungi	Bacteria, Chlorophyta, ascomycete Fungi, Ochrophyta

* see author list for affiliations.

The hub partners have defined the major scientific questions they wish to explore, and will source and identify specimens that will deliver answers. ASG follows an ethical code of sampling practice, avoiding overcollection and respecting local and international laws and protocols, especially as ASG will be sampling from endangered ecosystems and in some cases endangered species. The project participants are fully committed to the Convention on Biological Diversity Nagoya Protocols on Access and Benefit Sharing, and only samples where express permission has been obtained will be sourced and sequenced. Samples may come from the wild, from mesocosms and aquaria, from explant lab cultures or from culture collections.

Genome sequencing and assembly will be delivered by the Tree of Life programme at the Sanger Institute using pipelines being developed for the Darwin Tree of Life and other major biodiversity genomics projects. Genomes will be assembled, annotated and released openly through the European Bioinformatics Institute ( EMBL-EBI).

## Sequencing symbionts: from sample to openly accessible genome assembly

Each ASG Hub ( Table 1) has defined a set of taxa that it will sample for sequencing. We will sequence from single eukaryotic host specimens or clonal cultures rather than bulk samples whenever possible. While this can limit the mass of DNA and RNA available for sequencing, it has the very strong benefit of reducing allelic sequence complexity and enabling assembly. Importantly, we do not require that the symbiotic partners are separated before sequencing, as we will separate the host and symbiont genomes bioinformatically during assembly ( Challis *et al*., 2020).

Each sample is formally identified and associated with rich metadata describing its collection location and other environmental features. We collate and validate these metadata through the COPO biodiversity data brokering system. Samples are shipped to the Sanger Institute for long DNA and RNA extraction and sequencing, with particular focus on low-input methods. We are generating a combination of long read and long range genomic data. For long reads we primarily use the Pacific Biosciences Sequel IIe circular consensus sequencing approach to generate high fidelity (HiFi) reads in the 15 to 20 kilobase range, and include Oxford Nanopore Technologies long reads where needed. For long range data we use chromatin conformation capture sequencing (known as Hi-C). These long range data generate important information that link sequences within chromosomes and organelles in the multi-kilobase to megabase range and will allow us to disentangle genomes from different species. The joint transcriptome of the symbioses will be sampled using RNA-Seq, both on Illumina short read and Pacific Biosciences long read platforms.

We have strong expectations about what we should find in the sequence data, and what we should be assembling, but biology is full of exceptions and surprises and organisms taken from the wild are frequently found in association with other cobionts. Each symbiosis contains a community of genomes that can be viewed as a low complexity metagenome: the “host” genome and the genomes of its organelles (mitochondrion and in some cases plastid), the symbiont genome (which if it is eukaryotic contains one or more organellar genomes) and the genomes of other commensals and cobionts. We separate data into presumed organismal and organellar subsets and assemble each independently. First we identify taxonomically informative marker loci, such as small subunit ribosomal RNAs (organellar 12S, prokaryotic 16S and eukaryotic 18S), cytochrome oxidase I genes, and ribulose-1,5-bisphosphate carboxylase-oxygenase genes, in the HiFi reads and primary assembly. These tell us which taxa are likely to be present and thus which genomes we should expect to assemble. To separate the data we use intrinsic features (GC and tetranucleotide composition, read coverage, coding capacity), sequence similarity to known genomes, and Hi-C linkage information. Binning contigs and their constituent reads into distinct subsets facilitates complete assembly of each organismal and organellar genome ( Challis *et al*., 2020; Kumar & Blaxter, 2011). We aim to automate this cobiont identification and binning process, as it will be of utility in analyses of all Tree of Life genomes: many specimens harbour parasitic and other cobionts. Given 25- to 30-fold genome coverage in HiFi reads for each symbiont partner, we expect to generate primary assemblies with contig N50s in the multi-megabase range. The Hi-C data are used to scaffold these contigs into near-chromosomal pseudomolecules.

For each symbiotic system we will then curate the assemblies to improve accuracy ( Howe *et al*., 2021) with particular attention to correct scaffolding of nuclear chromosomes and circularisation of organellar and prokaryotic genomes, and identification of remaining complex and unresolvable repetitive regions (such as ribosomal RNA and centromeric repeats). We aim to achieve or exceed the latest Earth BioGenome Project ( Lewin *et al*., 2018) assembly standards. Curated assemblies and all raw data will be submitted to the European Nucleotide Archive ( ENA) ( Harrison *et al*., 2021) and from there to the rest of the International Nucleotide Sequence Database Consortium for immediate open release. The genomes will be annotated using the RNA-Seq transcriptomic data binned by species, and the annotations released openly. We have developed an ASG-specific data portal that collates all of the data generated by the project and promotes analysis. The Aquatic Symbiosis Genomics project relies on engagement and support from the whole of the Tree of Life production genomics team and of many colleagues who are participants in the ten Hubs. Each symbiotic system will be the subject of an open access publication, a Genome Note, that credits the full team that generated the assemblies, from collectors to annotators ( Threlfall & Blaxter, 2021).

## Building an aquatic symbiosis genomics community

The ASG project aims to generate a lasting resource in terms of the ~1000 genomes involved in ~500 symbiotic systems. To ensure this resource results in a flourishing ecosystem of postgenomic research, we are building community and expertise through a parallel programme of training and mentoring in genomics and bioinformatics. In collaboration with Wellcome Connecting Science and The Carpentries, the ASG project will deliver intensive and extensive collaborative training and investigative informatic analysis of symbiont genomes, to build collective genomics and bioinformatics capacity in the symbiosis community. Training will include core informatics, coding, and reproducible science, as well as deeper analytical dives into co-evolving genomes, detailed genome annotation, and prediction of the metabolic underpinnings of symbiotic cooperation.

Just as reefs built by corals and their symbiotic algae allow an exuberant and diverse ecology to thrive, the ASG project will build a lasting genomic foundation for flourishing and diverse analyses of symbioses. Many bony coral reef fish species have a pelagic early life history, their larvae spending their first weeks in the open ocean ( Leis & McCormick, 2002). These may be recruited back to the reef because they can hear and smell it: the chatter generated by a healthy reef attracts, recruits, and builds the reef community ( Gordon *et al*., 2019). Much like a healthy reef, our hope is that the high-quality genomes we produce will generate the chatter that attracts new researchers and provides a foundation for growth of fundamental research on the nature of symbiosis and conservation of habitats where symbioses abound.

## Data Availability

No data are associated with this article. ASG data will be released openly in the European Nucleotide Archive.
