# Supplementary material for: The Aquatic Symbiosis Genomics Project: probing the evolution of symbiosis across the Tree of Life
Source: Wellcome Open Res. 2024 Jun 10;6:254. Originally published 2021 Oct 6. [Version 2] doi: 10.12688/wellcomeopenres.17222.2 (PMC12117321; doi:10.12688/wellcomeopenres.17222.2)
Supplement: Peer review history

# Reviewer report: Reviewer response for version 2

**DOI:** 10.21956/wellcomeopenres.24685.r122605

**Published:** 2025-06-03

**Authors:** Joseph B. Kelly

**Affiliations:** 1 University of Konstanz, Konstanz, Germany

It is no difficult argument to make that the efforts of the ASG Project will provide an invaluable resource that will help the scientific community understand how symbiosis evolves. As evident in their taxonomic sampling scheme, the ASG Project also fills many gaps in the tree of life regarding genomic resources, and will therefore enable the identification of broadly-applicable biological principals. Given the importance of the genome assemblies being produced, I recommend that attention be given to the following points.

**1.** “Importantly, we do not require that the symbiotic partners are separated before sequencing, as we will separate the host and symbiont genomes bioinformatically during assembly ( Challis *et al*., 2020).”

**Comment:** Please also provide a counterpoint detailing what the shortcomings and possible risks are using this approach, and how they will be mitigated.

**2. **“First we identify taxonomically informative marker loci, such as small subunit ribosomal RNAs (organellar 12S, prokaryotic 16S and eukaryotic 18S), cytochrome oxidase I genes, and ribulose-1,5-bisphosphate carboxylase-oxygenase genes, in the HiFi reads and primary assembly. These tell us which taxa are likely to be present and thus which genomes we should expect to assemble.“

**Comment:** I would caution that the use of marker loci to guide the assemblies might be confounded in cases where you have multiple closely-related symbiont strains that may not yet have diverged at these loci. How will these cases be handled?

**3.** “To separate the data we use intrinsic features (GC and tetranucleotide composition, read coverage, coding capacity), sequence similarity to known genomes, and Hi-C linkage information.“

**Comment: **How will this pipeline be benchmarked within each major taxonomic clade?

**4.** "For each symbiotic system we will then curate the assemblies to improve accuracy ( Howe *et al*., 2021) with particular attention to correct scaffolding of nuclear chromosomes and circularisation of organellar and prokaryotic genomes, and identification of remaining complex and unresolvable repetitive regions (such as ribosomal RNA and centromeric repeats)."

**Comment: **Please provide details regarding what the curation process will entail.

Does the article adequately reference differing views and opinions?

Partly

Are all factual statements correct, and are statements and arguments made adequately supported by citations?

Yes

Is the Open Letter written in accessible language?

Yes

Where applicable, are recommendations and next steps explained clearly for others to follow?

Partly

Is the rationale for the Open Letter provided in sufficient detail?

Yes

Reviewer Expertise:

Bioinformatics, comparative genomics.

I confirm that I have read this submission and believe that I have an appropriate level of expertise to confirm that it is of an acceptable scientific standard.

# Reviewer report: Reviewer response for version 2

**DOI:** 10.21956/wellcomeopenres.24685.r122604

**Published:** 2025-05-27

**Authors:** Senjie Lin

**Affiliations:** 1 Department of Marine Sciences, University of Connecticut, Storrs, Groton, USA

The Letter effectively captures the scope of the Aquatic Symbiosis Genome Project, the diversity of symbiotic systems involved, and the anticipated outcomes. My main suggestion is to include more detail on the expected range of data quality or completeness. For example, it would be helpful to note that for species where high-molecular-weight (HMW) DNA suitable for long-read sequencing is not available, only raw or fragmented data may be produced, whereas for others, near-complete chromosomal assemblies will be achieved. Additionally, offering guidance on how readers should interpret the results and suggestions for improving dataset quality would further enhance the manuscript’s utility.

 I also have a couple of specific comments:

- **Regarding Table 1:** While I understand that listing all organisms involved may not be feasible, I recommend including as complete a list of **phyla** as possible. I noticed that a euglenid host with bacterial endosymbionts and a dinoflagellate with algal endosymbionts appear to be missing from the hub led by Joe Lopez.
- **Data access:** Adding links to data repositories mentioned in the Letter would be highly beneficial for readers interested in accessing and exploring the datasets.

Does the article adequately reference differing views and opinions?

Yes

Are all factual statements correct, and are statements and arguments made adequately supported by citations?

Yes

Is the Open Letter written in accessible language?

Yes

Where applicable, are recommendations and next steps explained clearly for others to follow?

Partly

Is the rationale for the Open Letter provided in sufficient detail?

Yes

Reviewer Expertise:

Ecological genomics of algae and protists

I confirm that I have read this submission and believe that I have an appropriate level of expertise to confirm that it is of an acceptable scientific standard.

# Reviewer report: Reviewer response for version 2

**DOI:** 10.21956/wellcomeopenres.24685.r122598

**Published:** 2025-05-27

**Authors:** Raúl A. González-Pech

**Affiliations:** 1 The Pennsylvania State University - University Park Campus, University Park, Pennsylvania, USA

This open letter presents the ambitious effort being done by the Aquatic Symbiosis Genomics Project to generate high-quality genomic data for about 500 systems. There is no doubt about the value of the resources this initiative is generating, which will advance the field of symbiosis to new frontiers.

 I have personally being tracking this project over the years and have even been tangentially involved with the sequencing of a few samples by providing material and advising in the data generation. I congratulate the team for pushing such a huge enterprise and for their achievements thus far. While I do not have any major comments, there are two minor concerns I have that might be worth expanding on in the text:

 1. Taking into consideration idiosyncratic genomic features of each of the systems for tailored data generation and bioinformatic analyses. For example, dinoflagellates (including coral symbionts) have a specific gene architecture (refer 1) that needs to be considered when doing gene prediction (refer 2). Because of this, people from ASG should consult with experts of each system to ensure optimal genomic data generation. I know this has been done to certain degree, but it could be taken further. A few words about this might be worth mentioning.

 2. Some of the symbionts might have complex systematics and their taxonomy (e.g., species designation) might not fully established. It would be useful to know how ASG is handling these cases.

 More punctual suggestions:

 - The "The genomics of symbiosis" section doesn't really touch on genomics. I suggest either changing the name of the section to something more generic (like "Background") or expanding on how genomics play an important role in understanding biology and evolution of symbiotic systems.

 - Following up on the previous point, while the authors mention a few examples of aquatic symbiotic systems, questions and directions that could be explored with the data being generated remain vague. I suggest, as previous reviewers have, mentioning a few more specific examples of topics that could be explored. A useful resource for this could be a review we recently published on marine holobionts (refer 2).

 - Single celled eukaryotes in the last sentence of the genomics of symbiosis sections should have a hyphen (single-celled eukaryotes).

 - Consider changing "Sequencing symbionts: from sample to openly accessible genome assembly" to "Sequencing symbionts: from sample to openly accessible genomic data".

 Other than those minor comments, I endorse indexing.

Does the article adequately reference differing views and opinions?

Partly

Are all factual statements correct, and are statements and arguments made adequately supported by citations?

Partly

Is the Open Letter written in accessible language?

Yes

Where applicable, are recommendations and next steps explained clearly for others to follow?

Partly

Is the rationale for the Open Letter provided in sufficient detail?

Yes

Reviewer Expertise:

Symbiodiniaceae, coral symbiosis, coral holobiont, comparative genomics, evolutionary genomics

I confirm that I have read this submission and believe that I have an appropriate level of expertise to confirm that it is of an acceptable scientific standard.

## References

[1] : Draft assembly of the Symbiodinium minutum nuclear genome reveals dinoflagellate gene structure. *Curr Biol* .2013;23(15) : 10.1016/j.cub.2013.05.062 1399-408 10.1016/j.cub.2013.05.062 23850284

[2] : Evidence That Inconsistent Gene Prediction Can Mislead Analysis of Dinoflagellate Genomes. *Journal of Phycology* .2020;56(1) : 10.1111/jpy.12947 6-10 10.1111/jpy.12947 PMC706500231713873

[3] : The Evolution, Assembly, and Dynamics of Marine Holobionts. *Ann Rev Mar Sci* .2024;16: 10.1146/annurev-marine-022123-104345 443-466 10.1146/annurev-marine-022123-104345 37552896

# Reviewer report: Reviewer response for version 2

**DOI:** 10.21956/wellcomeopenres.24685.r122603

**Published:** 2025-05-27

**Authors:** Alison L. Gould

**Affiliations:** 1 Temple University, Philadelphia, Pennsylvania, USA

This article presents an exciting global collaboration to generate high quality reference genomes for representative aquatic symbioses across the tree of life. With respect to the questions above, I found the article adequately references differing views and opinions, all factual statements are correct, the statements and arguments made are adequately supported by citations, and it is written in accessible language.

 I do think the rationale for the Open Letter is provided in sufficient detail, but your overall motivation for producing these reference genomes is somewhat lacking. You say this initiative will "Transform symbiosis research" in the header of one section but don't provide much support for how it will do so. This section instead provides details on the logistics and design of your hub. It would be helpful to include here some more details on how will the research community will benefit from having access to these genomic resources. In what ways is this going to "transform" symbiosis research?

 Other than this comment, I have a few minor suggestions.

 The figure legend could use more detail. I see that you updated the branch/text colors in the Archaeplastida to green and red but don't explain what the two different colors indicate. I suggest clarifying this point in the legend text.

 There is some inconsistency in the tense used throughout, specifically in the Sequencing Symbionts section. The section begins as if this is work to be done and then switches to present tense in the binning sequences paragraph. I recommend choosing one tense and maintaining throughout.

Does the article adequately reference differing views and opinions?

Yes

Are all factual statements correct, and are statements and arguments made adequately supported by citations?

Yes

Is the Open Letter written in accessible language?

Yes

Where applicable, are recommendations and next steps explained clearly for others to follow?

Yes

Is the rationale for the Open Letter provided in sufficient detail?

Partly

Reviewer Expertise:

Microbial symbiosis, marine science

I confirm that I have read this submission and believe that I have an appropriate level of expertise to confirm that it is of an acceptable scientific standard.

# Reviewer report: Reviewer response for version 1

**DOI:** 10.21956/wellcomeopenres.19032.r47364

**Published:** 2021-12-15

**Authors:** James Bernot

**Affiliations:** 1 Department of Invertebrate Zoology, Smithsonian National Museum of Natural History, Washington, DC, USA

This open letter presents a global genomics project focused on sequencing the genomes of 500 aquatic organisms comprising multiple lineages of protists, algae, fungi, and invertebrates, along with their symbionts. Collectively these hosts and symbionts encompass an enormous swath of the tree of life. It is therefore an ambitious and challenging project which nonetheless holds great promise, especially in light of its large, international team with diverse expertise in a variety of organismal systems, genomic techniques, and bioinformatic approaches. This project is of particular interest given that increased attention on microbiome research in recent years has revealed diverse interactions between host and symbionts. Clearly, genome biology is key to understanding the complexities of this interacting holobiome.

 In this paper, the authors describe their target hosts and symbionts, which they group into 10 project hubs, each with one to three project leads as currently designed. The manuscript presents the planned sequencing methods, which combine Illumina short reads, PacBio and Nanopore long reads, and Hi-C chromatin conformation data – the best practices in modern genome sequencing. The authors state they will couple the genomic sequencing with short and long read RNA-Seq, which will be crucial for annotating the genomes and holds promise for uncovering functional interactions between members of each host-symbiont community. The paper also briefly outlines the author’s bioinformatic approach to separate and assemble the host-symbiont metagenomes, which has several notable strengths including the ability to assemble difficult-to-culture organisms and characterize previously unknown symbionts.

 Like other large-scale genome sequencing initiatives, the major test of this project will be whether or not it can maintain the sustained, long-term effort needed to complete such an ambitious goal. If successful, this work will be of great interest to the biological science community, not only to those studying the target organisms, but also anyone interested in the microbiome, the evolution of symbioses, and the evolution of eukaryotic life itself.

 I have a few minor comments the authors may want to consider:

- While the project is clearly exploratory by design, the paper could be improved by the addition of some research questions the team wants to explore or hypotheses they hope to test.
- The hubs each present their major host and symbiont taxa, but what approaches will be used to select other symbiont taxa to characterize and analyze in detail, especially in cases of diverse host-symbiont associations? While characterizing known, ecologically important symbionts appears to be the priority of the project, it could be valuable to establish objective thresholds for targeting other symbionts to focus efforts on (for example, perhaps the number of gDNA reads as a proxy for symbiont abundance, or the number of RNA-Seq reads aligning to a genome as evidence of transcriptomic activity). Some objective guidelines could help avoid biasing the project towards those symbionts with better characterized host interactions and increase the possibility of elucidating new interactions.
- Work on mycorrhizal fungi, coral and squid symbionts, and others has shown that host-symbiont interactions can vary drastically across environmental conditions – the same host-symbiont species pair can be commensal, mutualistic, or parasitic depending on the environment. While the proposed framework will build a valuable foundation for understanding host-symbiont genomics, a more holistic view of these interactions requires understanding how relationships may change in different ecological contexts. Have the authors considered exploring any of these relationships under different environmental conditions? The possibility of drastically different interactions under varying ecological conditions lends addition importance to the author’s proposal to collect rich environmental metadata, especially because it may not be initially apparent which environmental variables are important to the host-symbiont interactions.
  Some examples of varying host symbiont-interactions that might be of interest:
  Bronstein (2001) ^1^ and Grman (2012) ^2^
- The proposed PacbBio gDNA and RNA-Seq may pose potential challenges. First, while sequencing costs continue to decrease, is it currently feasible to complete the proposed PacBio gDNA and RNA sequencing for each host, especially in the beginning of the project when these costs are still quite high (or is this more so an ideal the project aspires to)? Second, are the PacBio input requirements feasible for most of the proposed taxa? Perhaps the host taxa will have enough genetic material, but I can imagine challenges obtaining reasonable read depth for symbionts when the community is diverse and input material limited. Have the authors made estimates of necessary read depth for symbiont genome and transcriptome assembly?
- Coordinated, large scale efforts such as those described here hold great promise for pushing science forward in major ways, but also pose a number of challenges such as accountability for project goals, timely reporting requirements, transparency in progress, and objective measures of success. Towards this end, I credit the authors for having already developed a data portal and status tracking system to increase transparency and expedite access to research data. Any additional efforts to expound on timelines, publication strategies, progress updates, etc. would be beneficial.

In summary, this is a large-scale genomics project with ambitious goals that holds great promise for improving our understanding of host-symbiont interactions across extremely diverse aquatic taxa. It is of broad interest to the scientific community, and I for one look forward to seeing future publications on the proposed work.

Does the article adequately reference differing views and opinions?

Yes

Are all factual statements correct, and are statements and arguments made adequately supported by citations?

Yes

Is the Open Letter written in accessible language?

Yes

Where applicable, are recommendations and next steps explained clearly for others to follow?

Yes

Is the rationale for the Open Letter provided in sufficient detail?

Yes

Reviewer Expertise:

Evolutionary biology, genomics, bioinformatics, phylogenetics, taxonomy

I confirm that I have read this submission and believe that I have an appropriate level of expertise to confirm that it is of an acceptable scientific standard.

## References

[1] : The exploitation of mutualisms. *Ecology Letters* .2001;4(3) : 10.1046/j.1461-0248.2001.00218.x 277-287 10.1046/j.1461-0248.2001.00218.x

[2] : Plant species differ in their ability to reduce allocation to non-beneficial arbuscular mycorrhizal fungi. *Ecology* .2012;93(4) : 10.1890/11-1358.1 711-8 10.1890/11-1358.1 22690621

# Reviewer report: Reviewer response for version 1

**DOI:** 10.21956/wellcomeopenres.19032.r46287

**Published:** 2021-10-18

**Authors:** James Davis Reimer

**Affiliations:** 1 Graduate School of Engineering and Science, University of the Ryukyus, Nishihara, Japan

**Overview:**

 A solid statement paper explaining the rationale and general outline of the ambitious Aquatic Symbiosis Genomics Project, this letter is well put together and provides a general overview of the framework and research to be done in this project. I have only some minor comments that need to be addressed. Some of my comments are scientific, while others are related to the writing style and grammar.

**Major concerns:**

 None.

**Minor comments:**

1. Please note for the question “Does the article adequately reference differing views and opinions?” I have answered “no” but do not think that this question is relevant for this paper; our overall understanding of the importance of symbioses is quite clear.
2. Regarding the question “Are all factual statements correct, and are statements and arguments made adequately supported by citations?” I have answered partly, as there are one or two areas where references or additional references are needed (see below for details).
3. This comment is more at the discretion of the authors, but in the Abstract, the term “symbiosis” is used as a singular noun in two locations (“environmental importance of symbiosis” and “all who work on symbiosis”) where I would instead use the plural “symbioses”. I imagine this is a matter of style, and leave the final choice to the authors, but feel plural serves these sentences in question better. A similar comment can be made on page 5, in the sentence “…flourishing and diverse analyses of symbiosis.”
4. In the “genomics of symbiosis” section, the authors reference Weis (2019) when discussing the photosymbiotic association between cnidarians and Symbiodiniaceae, but think there are both previous and wider (from the viewpoint of ecology or coral reef science) references available. Authors do not need to replace this reference, but I would add at least one more here, the reference chosen is focused more on cell biology.
5. Following the comment directly above, the second half of this sentence needs a reference. (“create biodiversity hotspots which house upwards of 25% of all described species in the oceans”). I am also not certain if hotspots is the best term, or “hotspot” describing the Coral Triangle. I suppose if the authors also wish to emphasize biodiversity centers such as the Red Sea and the Caribbean then plural is OK here.
6. Figure 1 – why are the taxa Embryophyta and Streptophyta written in green? No explanation for this is given in the legend.
7. In the section “aquatic symbiosis genomics project will transform symbiosis research”, the third paragraph here (starting with “The hub partners…”) needs elaboration. I would at the least add in here active collaboration with local and regional collaborators, and also the deposit of specimens in appropriate museums or collections that have public access for all researchers.
8. Table 1: In two instances, the authors list “ *Symbiodinium*”, but I think they are referring to Symbiodiniaceae and not just the genus (see LaJeunesse *et al. *2018 Curr Biol). ^1^
9. Page 5 (of the PDF): I am not an expert on fish, but is “open spawners” the best term here? “Many of the fish that throng around coral reefs are open spawners, …”.
10. As well, in the sentence immediately following the one above, you state “They are recruited back to the reef because they can hear and smell it: …”. Is this true or can this be said for all of the “many of the fish that throng around coral reefs”? You may need to qualify this sentence to some degree.
11. I would change the “provides” in this sentence “Much like a healthy reef, our hope is that the high quality genomes we produce will generate the chatter that attracts new researchers and provides a foundation …” to “provide”.

Does the article adequately reference differing views and opinions?

No

Are all factual statements correct, and are statements and arguments made adequately supported by citations?

Partly

Is the Open Letter written in accessible language?

Yes

Where applicable, are recommendations and next steps explained clearly for others to follow?

Yes

Is the rationale for the Open Letter provided in sufficient detail?

Yes

Reviewer Expertise:

Symbiodiniaceae, coral reefs, cnidarians, field and molecular ecology

I confirm that I have read this submission and believe that I have an appropriate level of expertise to confirm that it is of an acceptable scientific standard, however I have significant reservations, as outlined above.

## References

[1] : Systematic Revision of Symbiodiniaceae Highlights the Antiquity and Diversity of Coral Endosymbionts. *Current Biology* .2018;28(16) : 10.1016/j.cub.2018.07.008 2570-2580.e6 10.1016/j.cub.2018.07.008 30100341

# Response

**Published:** 2024-05-08

**Authors:** Tree of Life Team Sanger

**Affiliations:** Wellcome Trust Sanger Institute, UK

**Response from the author team:** Thank you for the helpful comments. We have made the following changes to address your concerns:

- In the abstract and elsewhere in the article: we have changed the term “symbiosis” to the plural “symbioses” where appropriate.
- In the “ **Genomics of symbiosis**” section, we have added an additional reference as recommended.
- We have rephrased slightly and added a reference for the text concerning “create biodiversity hotspots which house upwards of 25% of all described ocean species”.

- In the legend for Figure 1 we have added an explanation for using red and green fonts to indicate the taxa with primary plastids that subsequently spread to other taxa.
- “In the section “The ASG project will transform symbiosis research”, the third paragraph here (starting with “The hub partners…”) needs elaboration.” We have added a link to a description of the hub partners.

- In Table 1 we have replaced the genus name “Symbiodinium” with the family Symbiodiniaceae referring to the family.
- We have rephrased and provided references for the text “Many of the fish that throng around coral reefs are open spawners, …”.
- Also in this paragraph, we have corrected the word provides to ‘provide’ in “Much like a healthy reef, our hope is that the high-quality genomes we produce will generate the chatter that attracts new researchers and provides a foundation for growth of fundamental …”
